# Copper Tolerance and Accumulation on *Pelargonium graveolens* L’Hér. Grown in Hydroponic Culture

**DOI:** 10.3390/plants10081663

**Published:** 2021-08-12

**Authors:** Antonios Chrysargyris, Rita Maggini, Luca Incrocci, Alberto Pardossi, Nikolaos Tzortzakis

**Affiliations:** 1 Department of Agricultural Sciences, Biotechnology and Food Science, Cyprus University of Technology, Limassol 3603, Cyprus; a.chrysargyris@cut.ac.cy; 2 Department of Agriculture, Food and Environment, University of Pisa, 56124 Pisa, Italy; rita.maggini@unipi.it (R.M.); alberto.pardossi@unipi.it (A.P.)

**Keywords:** antioxidants, bioaccumulation, copper toxicity, hydroponics, translocation factor

## Abstract

Heavy metal contamination is a major health issue concerning the commercial production of medicinal and aromatic plants (MAPs) that are used for the extraction of bioactive molecules. Copper (Cu) is an anthropogenic contaminant that, at toxic levels, can accumulate in plant tissues, affecting plant growth and development. On the other hand, plant response to metal-induced stress may involve the synthesis and accumulation of beneficial secondary metabolites. In this study, hydroponically grown *Pelargonium graveolens* plants were exposed to different Cu concentrations in a nutrient solution (4, 25, 50, 100 μM) to evaluate the effects Cu toxicity on plant growth, mineral uptake and distribution in plants, some stress indicators, and the accumulation of bioactive secondary metabolites in leaf tissues. *P. graveolens* resulted in moderately tolerant Cu toxicity. At Cu concentrations up to 100 μM, biomass production was preserved and was accompanied by an increase in phenolics and antioxidant capacity. The metal contaminant was accumulated mainly in the roots. The leaf tissues of Cu-treated *P. graveolens* may be safely used for the extraction of bioactive molecules.

## 1. Introduction

Copper (Cu) is an abundant transition metal of the lithosphere that is considered a relevant anthropogenic contaminant, as large amounts of this element have been released into the environment over the past decades [1,2]. In addition to the environmental impact of mining and smelting operations, the extensive application of Cu-containing fertilizers, pesticides and fungicides in agricultural practices has contributed to water body and soil contamination [1,2,3,4]; therefore, agricultural soils are particularly exposed to pollution by this contaminant. For example, Chen et al. [5] reported that in China, over 16% of agricultural soil is contaminated by heavy metals, and 2% is polluted by Cu only. Among heavy metals, Cu is often the only contaminant in vineyards, where it is extensively used against downy mildew [1,2,3,4]. According to the European Council Directive 86/278/EEC [6] on the protection of the environment, the permitted Cu concentration in agricultural soils amended with sewage sludge is 50–140 mg kg^−1^ for pH values in the range 6–7. For uncontaminated soils, Kabata-Pendias and Szteke [7] indicated a Cu concentration range of 1–140 mg kg^−1^, depending on soil texture; the same authors reported that soil Cu concentrations in the range 25–40 mg kg^−1^ may be toxic to plants below pH 5.5, as Cu availability increases with soil acidity. 

In nature, Cu commonly exists in the elemental metal form or as Cu^+^ or Cu^2+^ ions, although the oxidation states +3 and +4 can also be found [8]. Due to its redox properties, Cu at low concentration has a fundamental biological role for all living organisms, taking part in several metabolic reactions [9,10]. In higher plants, Cu is an essential micronutrient that is necessary for normal growth and development [11], being involved in mineral nutrition and electron transfer reactions that occur in vital processes, such as respiration and photosynthesis, chlorophyll and primary metabolites biosynthesis, or the scavenging of radicals [4]. The Cu concentration range that is considered normal for plants ranges from 2–5 to 30 mg kg^−1^ dry weight (DW) [12,13], while at higher concentrations Cu can cause toxicity symptoms [14]. Toxic levels of Cu in plants can impair biochemical reactions, affect gas exchanges, reduce plant growth [15,16]. Plants grown in Cu-polluted soils undergo oxidative stress and accumulate reactive oxygen species (ROS) [17], which induces the activation of antioxidant enzymes and the biosynthesis of antioxidant molecules. Cu toxicity has also been associated with an increased content of proline in plant tissues [18,19].

To counteract the effects of metal toxicity, plants have developed tolerance mechanisms such as metal complexation, storage in vacuoles, precipitation in cell walls, and downregulation of metal transporters via the plasma membrane [20,21]. On the other hand, plant species capable of effectively up taking heavy metals from the soil and accumulating these contaminants in tissues generally show a high translocation rate from roots to shoots. These species can be profitably used for phytoremediation through the removal of toxic metals from the soil, which represents a green and cost-effective strategy for the amelioration of marginal lands [22,23]. According to recent literature [2], about 500 species are currently used for the phytoremediation of metal polluted soils. Conversely, the accumulation of toxic metals by plant species that are employed for human usage represents a serious threat for the consumers safety and has become a health concern worldwide. Medicinal and aromatic plants (MAPs) are typically used in the food, pharmaceutical and cosmetic industries as a natural source of biologically active compounds [24], and are increasingly cultivated on a commercial scale to sustain the expansion of the market demand. Contamination of the plant material is a major health issue concerning the commercial production of MAPs [25]. On the other hand, the physiological markers of plant response to metal-induced stress are often beneficial bioactive secondary metabolites, mainly antioxidants such as phenolic compounds or essential oils constituents [26,27,28,29].

The *Pelargonium* genus in the family Geraniaceae comprises several hundreds of aromatic species, distributed worldwide in subtropical and temperate regions [30]. The essential oil from *Pelargonium* spp. is among the top 20 essential oils used all over the world [31] due to its well-known bioactive properties [32,33,34]. In addition, the pharmacological activity of *Pelargonium* spp. is attributed also to phenolic constituents such as flavonoids and hydroxycinnamic acid-derivatives [35]. *Pelargomium* spp. are tolerant to toxicity by heavy metals and have been successfully applied as hyperaccumulators for several metal contaminants [36], including Cu [37]. Particularly, *Pelargomium graveolens* L’Hér., popularly known as rose-scented geranium, has been reported by several authors as a good candidate for phytoremediation; in addition, the effect of heavy metals on the yield and quality of its essential oil has been widely investigated [38,39,40]. However, in recent years the pharmacological activity of *P. graveolens* has been increasingly linked also to the leaf content and composition of the pool of antioxidant phenolics [27,41,42,43,44], and much less is known about the influence of heavy metals on the concentration of these compounds. Therefore, the aim of the present study was to verify the tolerance of *P. graveolens* to Cu toxicity and to test the hypothesis that Cu-induced stress could stimulate the synthesis of antioxidant phenolic constituents, thus improving the medicinal properties of this species. With these objectives, we evaluated the effects of Cu exposure in *P. graveolens*, in terms of metal translocation to different plant organs, plant growth, and synthesis of bioactive phenolic metabolites.

## 2. Results

### 2.1. Visible Injury and Plant Growth

The plants appeared healthy during the whole growing cycle. Typical toxicity symptoms, such as leaf chlorosis and necrosis, were not observed in Cu-treated plants. Two-way ANOVA revealed that sampling date (D) significantly (*p* < 0.05; *p* < 0.001) affected the number of leaves produced and total upper fresh biomass, while neither Cu nor the interaction of date x Cu (D x Cu) affected the plant height, leaf number and total upper fresh biomass and dry matter content (Table 1). Copper concentration in the nutrient solution affected pelargonium growth parameters (Table 1). Plants grown with ≥25 μM Cu in the nutrient solution produced lower number of leaves at 35 DAT (days after transplanting), but this effect did not persist at 49 DAT. Total upper fresh biomass (including leaves, petioles and stems) decreased at the highest Cu (100 μM Cu) levels compared with the plants grown at 25 μM or 50 μM Cu after 35 DAT. Dry matter content at 49 DAT increased in plants grown in ≥50 μM Cu compared to 25 μM Cu and control treatment.

**Figure 1:**
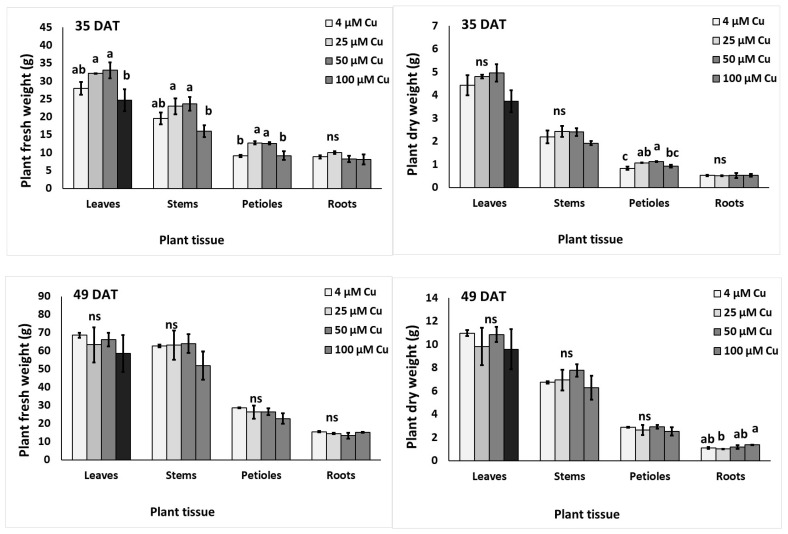
Effect of increasing copper (Cu) concentration (4–25–50–100 μM Cu^2+^) in the nutrient solution and sampling date after transplanting (DAT, 35 days and 49 days) on the fresh (FW; g plant^−1^) and dry weight (DW; g plant^−1^) of leaves, stems, petioles and roots respectively, of pelargonium plants grown hydroponically in perlite. Significant differences (*p* < 0.05) among Cu concentrations for each plant tissue are indicated by different letters; ns indicates non-significant. Error bars show SE (*n* = 6).

Looking at the fresh and dry biomass of individual plant organs, it was found that leaves and stems were increased at 35 DAT at 25–50 μM Cu compared to 100 μM Cu (Figure 1). Petiole fresh weight (FW) was also increased at 25–50 μM Cu compared with control or 100 μM Cu. Copper levels did not affect the root FW at 35 DAT. Following 49 DAT, leaf, stem, petiole and root FW were at similar levels (averages of 64.24, 26.13, 60.48 and 14.44 g, respectively), independent of the Cu concentration in the nutrient solution. Petiole dry matter content increased in 25–50 μM Cu, compared to the control treatment at 35 DAT. However, root dry matter content increased in 100 μM Cu compared to 25 μM Cu at 49 DAT.

### 2.2. Effects on Plant Physiology Attributes

Plants grown at the high Cu concentration of 100 μM Cu revealed higher stomatal resistance at both 35 and 49 DAT, compared to the control treatment (Table 2). Contrarily, chlorophyll fluorescence as measured by Fv/Fm (representing the maximum quantum yield of PSII), decreased at 100 μM Cu when compared to control and/or 25 μM Cu at 35 DAT and 49 DAT. The content of chlorophylls, as measured by chlorophyll a, chlorophyll b and total chlorophylls, did not change among the treatments at 35 DAT, but decreased at higher Cu levels (i.e., 100 μM Cu) at 49 DAT compared to the control and/or 25 μM Cu. 

Two-way ANOVA revealed that sampling date (D) significantly affected stomatal conductivity and chlorophyll fluorescence (*p* < 0.001); copper levels significantly affected stomatal conductance and chlorophyll a (*p* < 0.05), while the interaction of sampling date and copper (D x Cu) did not affect the examined physiological parameters (Table 2). 

### 2.3. Effects on Total Phenols, Flavonoids and Antioxidant Activity

**Figure 2:**
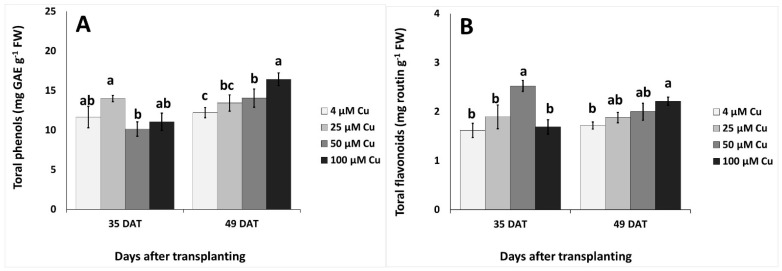

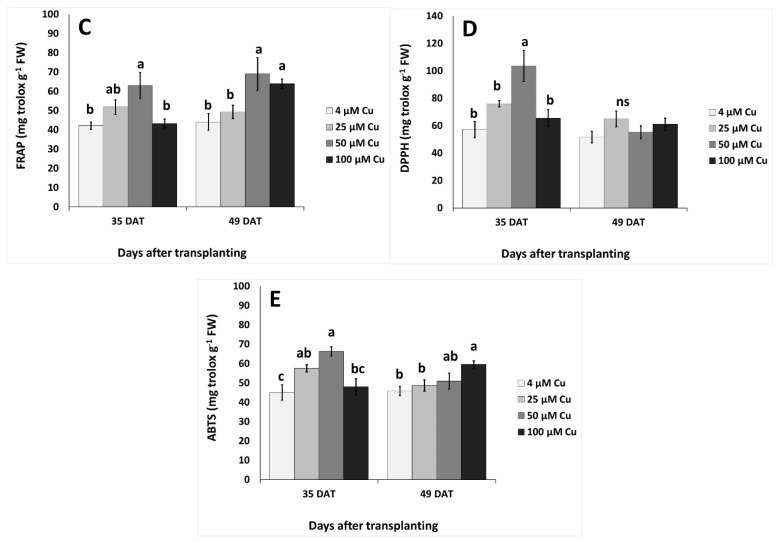
Effect of increasing copper (Cu) concentration (4–25–50–100 μM Cu^2+^) in the nutrient solution and sampling date after transplanting (DAT, 35 days and 49 days) on the leaf content of total phenols, total flavonoids and antioxidant activity in pelargonium plants grown hydroponically in perlite. (**A**) Total phenols, (**B**) total flavonoids, (**C**) FRAP (**D**) DPPH, and (**E**) ABTS. Significant differences (*p* < 0.05) among Cu concentrations at each sampling date are indicated by different letters; ns indicates non-significant. Error bars show SE (*n* = 4).

Two-way ANOVA revealed that sampling dates (35 vs. 49 DAT) significantly effected total phenols and DPPH (2,2-diphenyl-1-picrylhydrazyl) (*p* < 0.01), Cu levels significantly effected ABTS (2,2′-azino-bis(3-ethylbenzothiazoline-6-sulphonic acid) (*p* < 0.05) and flavonoids (*p* < 0.01), while the interaction of the sampling date x Cu effected ABTS and flavonoids (*p* < 0.05) and total phenolics (*p* < 0.01). The content of flavonoids and antioxidant activity (as assayed by ferric reducing antioxidant power; FRAP, DPPH, ABTS) revealed their highest values at 50 μM Cu, when compared with control and 100 μM of Cu, and differed significantly also from 25 μM Cu in the case of flavonoids and DPPH at 35 DAT (Figure 2). The content of total phenols and flavonoids, as well as antioxidant activity as assayed by FRAP and ABTS, revealed an increased trend as the Cu level increased at 49 DAT, with significant differences at the high Cu levels compared to the control treatment (Figure 2A–C,E). 

### 2.4. Plant Stress Indices 

**Figure 3:**
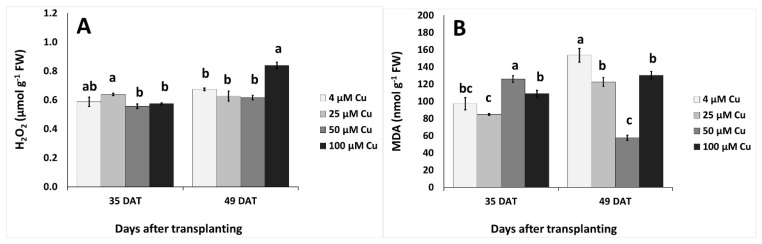
Effect of increasing copper (Cu) concentration (4–25–50–100 μM Cu^2+^) in the nutrient solution and sampling date after transplanting (DAT, 35 days and 49 days) on the leaf content of hydrogen peroxide (H_2_O_2_; (**A**)) and malondialdehyde (MDA; (**B**)) in pelargonium plants grown hydroponically in perlite. Significant differences (*p* < 0.05) among Cu concentrations at each sampling date are indicated by different letters. Error bars show SE (*n* = 4).

Two-way ANOVA revealed that sampling dates (35 vs. 49 DAT), Cu levels and their interactions significantly affected hydrogen peroxide (H_2_O_2_) and malondialdehyde (MDA) levels (*p* < 0.01, *p* < 0.001). Hydrogen peroxide levels increased at 25 μM Cu in comparison to the 50–100 μM Cu, but did not differ from the control at 35 DAT (Figure 3A). Following 49 DAT, H_2_O_2_ increased at 100 μM Cu compared to lower Cu levels and/or control. Lipid peroxidation (as assayed by MDA) increased at 50 μM Cu in comparison to higher or lower Cu levels at 35 DAT, while MDA decreased with ≥25 μM Cu compared to the control treatment (at 4 μM Cu) at 49 DAT (Figure 3B).

### 2.5. Copper Content in Plant Tissues

Two-way ANOVA revealed that sampling date (D) significantly affected AR, BAC-roots, BAC-stems, TF-leaves, TF-stems, TF-petioles (*p* < 0.001), and BAC-petioles (*p* < 0.05); Cu levels significantly affected BAC-roots, BAC-leaves, BAC-stems, BAC-petioles, TF-leaves, TF-stems, and TF-petioles (*p* < 0.001), while the interaction of sampling date and Cu (D x Cu) affected BAC-roots, BAC-stems, BAC-petioles, TF-leaves, TF-stems and TF-petioles (*p* < 0.001) (Table 3). The copper accumulation rate increased at 50 μM of Cu compared with the control at 35 DAT. All bioaccumulation coefficients and translocation factors for leaves, stems, petioles and roots were significantly decreased with ≥25 μM of Cu in the nutrient solution at 35 and 49 DAT (Table 3).

Regarding tolerance index, two-way ANOVA revealed that sampling date (D) significantly affected TI-petiole FW and TI-petiole DW (*p* < 0.001); copper levels significantly affected TI-total biomass, TI-stem FW and TI-petiole FW (*p* < 0.05); while the interaction of sampling date and Cu (D x Cu) affected only the TI-petiole FW (*p* < 0.05) (Table 4). Tolerance index values of plant growth were affected at 35 DAT, as TI increased at 25–50 μM Cu for leaf, stem and petiole FW and as a consequence of the plant total biomass when compared with 100 μM Cu in the nutrient solution (Table 4). Similarly, TI-leaf DW and TI-petiole DW were also increased at 20–50 μM Cu. The TI of leaf number was decreased with ≥25 μM Cu in the nutrient solution at 35 DAT. At 49 DAT, the TI for total biomass was increased with 25 μM Cu in the nutrient solution, while TI-root DW increased at 100 μM Cu when compared to ≤25 μM Cu (Table 4).

### 2.6. Responses of Other Nutrients 

**Figure 4:**
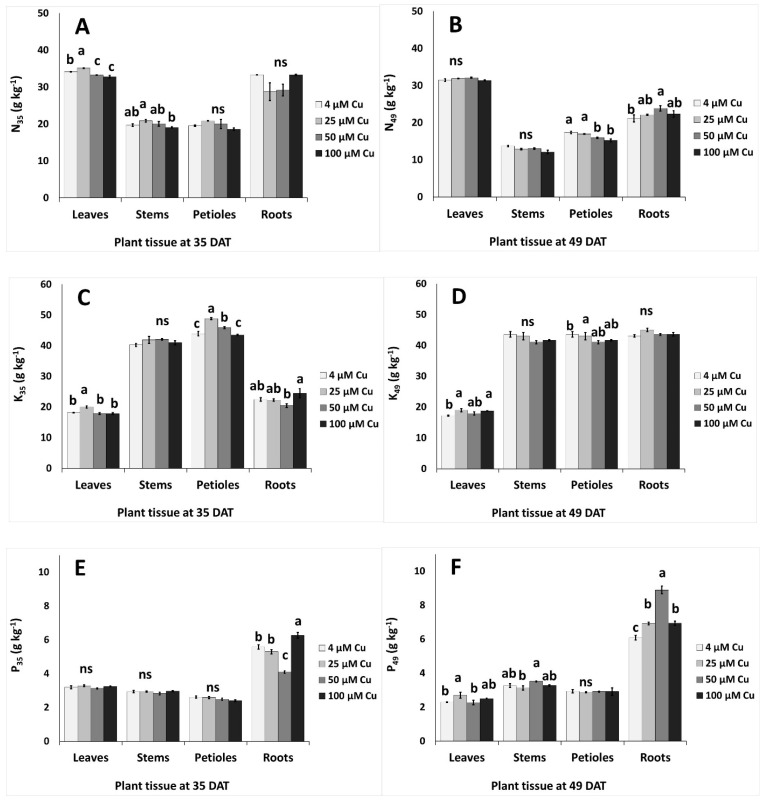

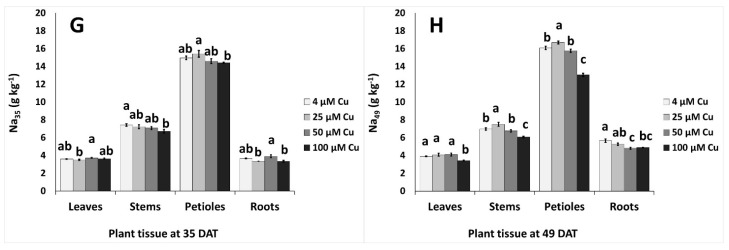
Effect of increasing copper (Cu) concentration (4–25–50–100 μM Cu^2+^) in the nutriment solution and sampling date after transplanting (DAT, 35 days and 49 days) on the content of macronutrients and sodium in different organs of pelargonium plants grown hydroponically in perlite. (**A,B**) Nitrogen–N, (**C,D**) potassium–K, (**E,F**) phosphorus–P, (**G,H**) sodium–Na. Significant differences (*p* < 0.05) among Cu concentrations at each sampling date are indicated by different letters; ns indicates non-significant. Error bars show SE (*n* = 4).

**Figure 5:**
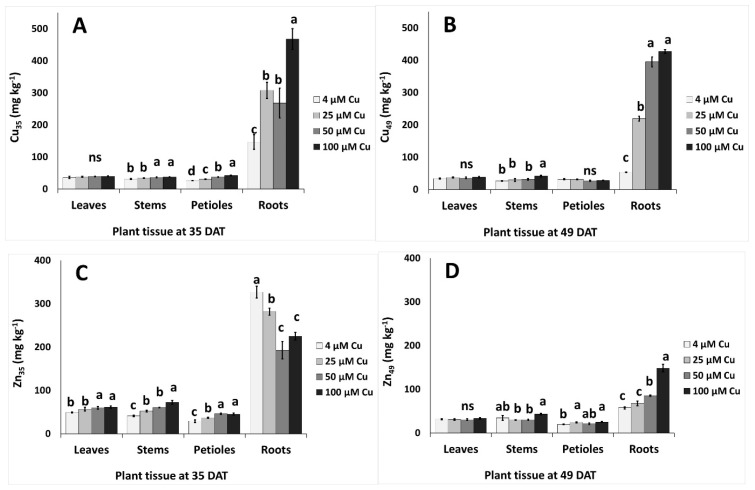
Effect of increasing copper (Cu) concentration (4–25–50–100 μM Cu^2+^) in the nutriment solution and sampling date after transplanting (DAT, 35 days and 49 days) on the content of micronutrients in different organs of pelargonium plants grown hydroponically in perlite. (**A,B**) Copper–Cu, and (**C,D**) zinc–Zn. Significant differences (*p* < 0.05) among Cu concentrations at each sampling date are indicated by different letters; ns indicates non-significant. Error bars show SE (*n* = 4).

The accumulation of nutrients in different plant organs (leaves, stems, petioles and roots) under different Cu levels at two sampling periods (35 and 49 DAT) is described in Figure 4 and Figure 5. At 35 DAT, the leaf content of N and K increased in 25 μM Cu and decreased or remained unaffected in ≥50 μM Cu compared to the control (Figure 4A,C). Stem N decreased at 100 μM Cu when compared with 25 μM Cu, however the N level in petioles and roots remained similar in plants grown with different Cu levels in the nutrient solution (Figure 4A). Leaf and stem N levels were similar at 49 DAT in all examined Cu levels in the nutrient solution (Figure 4B). Petiole K increased in 25–50 μM Cu compared to the control (Figure 4C). Phosphorus content in leaves, stems and petioles was unaffected by the Cu levels in the nutrient solution, while P in roots decreased at 50 μM Cu and increased at 100 μM Cu compared to the control treatment (Figure 4E). Increased P levels were found at 49 DAT in roots at 50 μM Cu (Figure 4F). Sodium accumulated more in petioles, compared to leaves, stems and roots, while Na content decreased at high Cu levels (Figure 4G,H). 

Copper accumulated in stems, petioles and roots as the Cu concentration increased in the nutrient solution; at 35 DAT, greater effects were observed in roots (2.2-fold increase at 100 μM Cu compared to the control treatment) (Figure 5A). A similar trend was found in Cu accumulation even at 49 DAT, but the increment in roots at 50–100 μM Cu was 6.9-fold greater compared with the control treatment (Figure 5B). Zinc accumulated more in leaves, stems, and petioles at 35 DAT as Cu levels increased in the nutrient solution, whereas Zn content decreased in roots with increasing Cu concentration in the nutrient solution (Figure 5C). However, the reverse was evidenced at 49 DAT, as Zn accumulated in roots following increases of Cu levels in the nutrient solution (Figure 5D).

### 2.7. Regression Analysis

Pearson’s correlation coefficients were determined between individual pairs of parameters associated with Cu uptake (leaf and root Cu concentrations), leaf antioxidant systems (content of total phenols and flavonoids, antioxidant capacity according to FRAP, DPPH and ABTS assays), and oxidative stress (H_2_O_2_ and MDA) (Table 5).

Leaf Cu concentration was positively correlated to the root content of the element at both sampling dates. The correlation coefficients between leaf or root Cu and the biochemical parameters were generally higher at 49 than 35 DAT, and in older plants all correlations were positive except those involving MDA content.

## 3. Discussion

Plant growth and development are regulated by plant physiology, which in turn is tightly linked to both environmental conditions and, in hydroponic cultivation, to the composition of the nutrient solution that is supplied to the plants [45,46]. For evaluation of the effects of Cu toxicity, the choice of appropriate Cu levels depends on the species being tested, the duration of the Cu treatment, and other growing parameters such as the pH of the nutrient solution. In this study, hydroponically grown plants of *P. graveolens* were exposed to Cu concentrations of up to 100 µM. Similar concentrations were tested in several species including Moso bamboo [5], maize [15], or *Carthamus tinctorius* L. [1]. Although higher Cu levels have been reported for turfgrass (120 µM) [16], and particularly for tomato (250 or 350 µM) [47,48], lower concentrations (up to 40 µM) were employed for both tree species [49] or vegetable crops [46].

In our experiments, all growth parameters were, of course, significantly higher at 49 than 35 DAT, with the only exceptions being plant height and total upper dry matter percentage, which were not affected by plant age. At 35 DAT, despite the lower number of leaves in Cu-treated plants than in the control, Cu concentrations up to 50 μM in the nutrient solution increased the fresh biomass production of the aerial part (Table 1); however, a relevant increase in dry matter was observed only for stem tissues, suggesting that the overall effect was partially due to increased water absorption. In contrast, at 49 DAT the fresh weight of the distinct aboveground plant organs did not change across treatments (Figure 1), while the increase in dry mass percentage above 50 μM Cu (Table 1) indicated a lower water content in those tissues. An increase in the percentage of dry matter was observed also in the leaf tissues of tomato plants grown in hydroponics, after 15 days exposure to 100–350 μM Cu concentrations [48]. The detrimental effect of high Cu levels in the root zone on biomass production was observed in several food crops [4], and in MAPs such as *Carthamus tinctorius* [1]. Similarly, in this work both fresh (Table 1) and dry (Figure 1) biomass production were tendentially lower in the 100 μM Cu treatment than the control at both sampling dates, although the difference was never significant. These results showed that *P. graveolens* is a species tolerant to Cu toxicity of up to 100 μM concentration, consistent with the tolerance indices toward metal stress reported in Table 4. For each plant organ, the values of the latter parameters were generally similar to those of the corresponding control. Toxic Cu concentrations well below 100 µM have been reported in the literature for several species. For example, Reichman et al. [50] reported that the highest Cu concentration in the nutrient solution without negative effects on plant growth was 35 μM for Cu-tolerant populations of *Silene cucubalus*; the no-effect threshold was about 5 μM for Cu-sensitive cultivars of mung bean, sweet potato and wheat, and was below 1 μM in Australian tree species such as ironbark, *Acacia holosericea*, and *Melaleuca leucadendra*. 

Plant age significantly influenced both stomatal resistance, which was much higher at 49 than 35 DAT for all the Cu treatments, and photosynthetic efficiency, expressed as chlorophyll fluorescence Fv/Fm, which was lower in older plants. At both sampling dates, increasing Cu concentrations in the nutrient solution interfered with the process of photosynthesis by increasing stomatal resistance and decreasing leaf chlorophyll fluorescence (Table 2). Along with the determination of stomatal resistance, the assessment of chlorophyll fluorescence is a key parameter in the rapid detection of response to physiological stress in higher plants; specifically, the Fv/Fm ratio is a physiological marker of photoinhibition of photosystem II (PSII) induced by stress conditions [51]. It has been reported that excess Cu can impair photosynthetic electron transport particularly at the PSII level, and Cu toxicity has been associated with quenching of variable fluorescence Fv [52]. The values of Fv/Fm reported in Table 2 remained within the typical range for healthy plants, that is 0.75–0.85 [53], and suggested that, despite a significant decline of the indicator at the 100 μM Cu concentration at both sampling dates, the function of the PSII reaction centers was preserved with all Cu treatments. Although plant age did not affect the content of chlorophylls, a significant decrease with increasing Cu concentration was observed at 49 DAT for these pigments. The above data could be reasonably interpreted as early indicators of Cu toxicity that became more severe with the duration of exposure; despite the effects of a possible photosynthetic imbalance this did not translate into a significant biomass decrease, or in the typical visible symptoms of toxicity such as leaf chlorosis [54]. Under impaired photosynthesis, plant metabolism is affected and one possible biochemical process that can be activated is the Mehler reaction, with formation of oxygenated molecules such as H_2_O_2_ [55]. This process is consistent with the significant increase in H_2_O_2_ concentration that was observed at 49 DAT in plants treated with 100 μM Cu (Figure 3A). 

It is generally acknowledged that excess Cu causes oxidative stress in plants [1,2,13,17]. However, due to the time course of the antioxidant response, the levels of stress indicators in plant tissues may undergo fluctuations. This could account for the significant effect of sampling date, Cu concentration, and their interaction on the observed contents of both H_2_O_2_ and MDA, and could also explain the contrasting results reported in the literature concerning the levels of ROS or MDA in several plant species [4]. The antioxidant activity of pelargonium at 35 DAT showed the same behavior across the Cu treatments (Figure 2C–E), regardless of the assay used for the determination (FRAP, DPPH or ABTS) and the stimulation of the plant antioxidant response was strictly related to the occurrence of lipid peroxidation, since a similar pattern was observed also for the concentration of MDA (Figure 3B). The content of total flavonoids followed the same trend (Figure 2B), suggesting that this class of compounds could play a key role in the pool of antioxidant molecules of *P. graveolens* that are involved in plant response to excess Cu in the early stages of exposure. Interestingly, the highest values of antioxidant power and flavonoid concentration were obtained with the 50 μM Cu treatment, which also resulted in the highest rate of Cu accumulation in plant tissues (Table 3). On the other hand, a different behavior was observed for total phenols (Figure 2A), whose amount, unlike the content of flavonoids, was affected also by the sampling date. These dissimilarities indicated that, along with flavonoids, other classes of phenolic compounds could contribute significantly to the pool of phenolics of this species. 

At 49 DAT, all parameters except DPPH scavenging capacity increased with Cu concentration (Figure 2), suggesting the occurrence of an effective dose-dependent response of the antioxidant system to excess Cu and a central role of phenolic compounds in the plant tolerance to Cu toxicity. These findings showed the effectiveness of high Cu concentrations in the nutrient solution in stimulating the synthesis and accumulation of beneficial antioxidant molecules in the plant tissues of *P. graveolens*. In addition to phenolics, other non-enzymatic antioxidant compounds could have an impact on plant response to Cu toxicity; for example, an increased content of proline has been observed in different species exposed to excess Cu [18,19]. In our experiments, the level of MDA decreased with Cu concentration in the nutrient solution up to 50 μM, showing that Cu-exposed *P. graveolens* could well counteract lipid peroxidation. Likewise, the leaf concentration of H_2_O_2_ was effectively controlled in up to 50 μM Cu. In contrast, the increase in the content of H_2_O_2_ at 100 μM Cu may indicate a less efficient plant response at this high concentration of the element (Figure 3A,B). 

The relationships among the indicators linked to Cu uptake and antioxidant response to Cu treatments are confirmed in the Pearson’s correlation table (Table 5). Leaf Cu content followed root content at both sampling dates, and, in younger plants, the content of flavonoids rather than the level of total phenols was strongly correlated with the antioxidant capacity as obtained using either the FRAP, DPPH or ABTS assays. However, at 35 DAT, Cu exposure did not elicit a marked response, as extremely weak relationships were evidenced between Cu levels in the tissues and all other biochemical parameters. The correlation coefficients between the concentrations of Cu and those of H_2_O_2_ and MDA also remained low at 49 DAT, suggesting that even older plants could efficiently prevent oxidative stress. On the other hand, higher values of the correlation coefficients were generally evidenced between root or leaf Cu levels and the other biochemical parameters (phenols or flavonoids content, or antioxidant capacity). Therefore, the results of the regression analysis are consistent with an initial antioxidant response to Cu toxicity at 49 DAT.

The TF and BAC indexes are important parameters for the evaluation of plant phytoremediation potential. In hyperaccumulator species, both parameters are greater than 1; in contrast, in our study only the BAC factor was higher than 1, indicating that *P. graveolens* acted as a Cu excluder. According to Saleem et al. [13]. Cu excluders, which have a low potential for metal extraction, could be effectively employed for phytostabilization. The BAC and TF indexes showed significant decreases with increasing Cu concentration in the nutrient solution at both sampling dates (Table 3). At low concentrations (up to 25 μM), young plants accumulated more Cu in root tissues and showed a much larger variation of the root BAC index among the Cu treatments than those sampled at 49 DAT. However, the decrease of root BAC values at both sampling dates indicated a strong inhibition of Cu uptake as the concentration of the element in the nutrient solution increased. A much larger variation was observed for the BAC index of the aerial parts, which decreased more than 10-fold in all aboveground tissues during the whole growing cycle, suggesting a synergistic effect of reduced Cu uptake and reduced element translocation in Cu-treated plants. This outcome was confirmed by lower TF values in the Cu treatments as compared to the control. Although in the latter treatment the TF in the aboveground tissues was higher in older plants, Cu translocation was markedly limited with increasing Cu concentration at both sampling dates. Therefore, the observed tolerance of *P. graveolens* to Cu toxicity was both due to the plant’s ability to exclude Cu from the leaf tissues by limiting translocation to the aerial parts, and to an efficient antioxidant system. A similar behavior was observed in *Solanum cheesmaniae* subjected to Cu stress [56]. This effect was further evidenced by the results shown in Figure 5A,B, as Cu accumulated particularly in root tissues independent of the concentration of the nutrient solution. In contrast, the stems were only slightly affected by the 100 μM Cu treatment, and the leaf tissues were totally unaffected. 

According to Lange et al. [57], most Cu-tolerant species act as Cu excluders, with very low Cu translocation from root to shoots. Contrarily, Chen et al. [5] reported on 25 plant species identified as Cu hyperaccumulators and provided literature data concerning tolerant and accumulator species, with leaf Cu content ranging from 45 to 596 mg/kg DW and root content ranging from 33 to 3768 mg kg^−1^ DW. In plants, Cu uptake is generally dependent on the species, plant organ, concentration in the growing medium, and the time of exposure. For example, according to Adrees et al. [4], maize plants exposed for six days to 100 μM Cu in hydroponics accumulated 1070 and 56 mg kg^−1^ DW in roots and shoots, respectively; the same species was reported to accumulate 7790 mg kg^−1^ DW in the roots after 15 days treatment with 80 μM Cu. Chen et al. [5] reported that in hydroponically grown *Moso bamboo* with 100 μM Cu in the nutrient solution, the Cu content in leaf and root tissues were, respectively, 24 and 417 mg kg^−1^ DW after 15 days, and 91 and 809 mg kg^−1^ DW after 30 days exposure. Saleem et al. [13] reported that pot-grown flax accumulated Cu mainly in the root tissues after 35 days cultivation, while the contaminant was accumulated mainly in the shoots in mature plants (105–140 days). In our experiments, despite a dose-dependent Cu accumulation in the roots of up to 468.14 mg kg^−1^ DW, Cu content in the leaf tissues remained at the same level as the control both at 35 and 49 DAT, further characterizing *P. graveolens* as a Cu excluder species. 

In a very recent paper, Tschinkel et al. [58] reported that the permitted concentration of impurities for drug substances and excipients set by the United States Pharmacopoeia Convention (USP) is 300 mg kg^−1^. Additionally, according to a recent review from the European Food Safety Authority [59], the maximum residue level (MRL) for Cu compounds (Cu) in leaves and herbs for herbal infusions is 100 mg kg^−1^. These limits are much higher than the Cu concentrations that were found in the leaf tissues of *P. graveolens*, which were below 50 mg kg^−1^ DW (Figure 5A,B). Therefore, the leaves of Cu-treated *P. graveolens* plants had higher contents of antioxidants and, at the same time, the same Cu content of the control plants, and may be safely used in the pharmaceutical/herbal industry for the extraction of phenolic compounds and other beneficial constituents such as essential oils. Although the latter were not examined in this work, some authors showed that heavy metals had minimal impact on the quality of *P. graveolens* essential oil, even when the contaminant was partly translocated to the aboveground organs [38,39]. In general, despite a high translocation factor from the root system to the aerial parts being indispensable for species with edible roots, the opposite is preferable for plant species that are used for leaf tissues, like the one examined in this study. Considering the excess of Cu application in agriculture and the consequent contamination of soils and water bodies, selection of MAPs according to their tolerance and potential accumulation in the organs of interest becomes a crucial issue in managing the problem of Cu pollution and preserving the quality of plant materials. It is noteworthy to mention that expanded and unexpanded perlite have some properties that can favor the adsorption of metal ions, including Cu [60]. However, the strong root Cu uptake shown in Figure 5A,B showed that this microelement was available to the plants in all Cu treatments.

Copper had a strong influence on Zn uptake (Figure 5C,D). In the root tissues of younger plants, Zn content was inversely related to that of Cu; this indicated a competitive absorption mechanism for the two micronutrients, in agreement with what reported by Kabata-Pendias and Szteke [7]. The amount of Zn in the aerial parts at 35 DAT increased significantly across the Cu treatments, suggesting that higher Cu levels promoted Zn translocation to the stems. Conversely, Cu and Zn uptake followed the same trend in older plants, despite a much lower Zn accumulation at 49 than 35 DAT. A decrease of Zn uptake in plants exposed to excess Cu has been observed in several species [4]. The content of Na decreased with increasing Cu in all plant organs, especially at 49 DAT, in agreement with what reported by Chrysargyris et al. [49] for the roots of *Mentha spicata*. The opposite trend was observed by other authors in the leaves of *Vicia faba* [61] and the shoots of pistachio seedlings [62]. The addition of Cu to the nutrient solution generally did not have a significant influence on the uptake of the macronutrients N, P and K, which was confirmed the scarce effects observed on the biomass production (Table 1 and Figure 1). The only exception was root P content at 49 DAT, which was higher in Cu-treated plants than in controls (Figure 4E,F), in contrast with the results reported by Chrysargyris et al. [49] and Eskandari and Mozaffari [62]. According to Adrees et al. [4], although Cu supply generally affects mineral nutrition, the effect of this microelement on the uptake of other mineral nutrients is strongly dose-, time- and species-dependent. In addition, in polluted environments, Cu could interact with other heavy metal contaminants [9,26,51,63]. In general, we observed that the Cu treatments did not impair mineral nutrition and, overall, *P. graveolens* showed a high capacity to grow in Cu-enriched mediums of up to 100 μM. 

Further work is necessary to provide a deeper insight into the response of *P. graveolens* to Cu stress. For example, the effects of severe Cu exposure conditions could be investigated through an extension of the growing period beyond 49 DAT, or with Cu concentrations higher than 100 μM in the nutrient solution; additionally, a similar experiment could be carried out in open field, where Cu bioavailability is conditioned by soil properties; finally, the profiling of individual metabolites of interest under Cu stress could help in highlighting the effects of this element on the bioactive properties of *P. graveolens*. 

## 4. Materials and Methods

### 4.1. Plant Material and Cultivation Conditions

*Pelargonium graveolens* L’Hér. plants were selected for the present study, which was implemented at the experimental greenhouse of Cyprus University of Technology, in Limassol, Cyprus. Cuttings of 10 cm length were collected from mother plants (National Agricultural Department, Nicosia, Cyprus) and were grown in peat:perlite (4:1 *v*/*v*) substrate, in plastic seedling trays for 25 days, till roots formation. Plants at the stage of four-to-five leaves were transplanted in pots (one plant per pot; 1.5 L capacity) filled with expanded perlite and placed on plastic trays to achieve proper drainage (see Chrysargyris et al. [64]). Perlite properties have been described previously [65]. Plants were grown in an open (free drainage) hydroponic system and the drainage nutrient solution was available to plants through capillary suction. Plants were sampled at two different growth stages. 

Plants were initially grown with the application of a full-strength nutrient solution (electrical conductivity (EC) and pH of 2.1 mS cm^−1^ and 5.7, respectively) for 21 days. Nutrient solution composition was: NO_3_^−^-N = 15.00, K = 9.50, PO_4_^−3^-P = 1.80, Ca = 4.20, Mg = 1.63, SO_4_^−2^-S = 1.55 and Na = 1.85 mmol L^−1^, respectively; and B = 30.00, Fe = 35.05, Mn = 6.10, Cu = 4.00, Zn = 4.10, and Mo = 0.52 μmol L^−1^, respectively. The described above concentrations were obtained using mineral salts and chelate for iron with ethylenediamine-N-N’bis(2-hydroxy-4-methylphenylacetic) acid (6.5% Fe EDDHMA). After that period, plants were subjected to different Cu levels (treatments) in the nutrient solution, namely (i) 4 μM Cu (control); (ii) 25 μM Cu; (iii) 50 μM Cu; and (iv) 100 μM Cu (in the form of CuSO_4_). Plants were grown under Cu excess for additional 28 days (in total 49 days after transplanting, DAT). A total of 96 plants were used (4 Cu levels × 2 sampling periods × 12 replicates). 

### 4.2. Plant Growth and Physiological Measurements

Plant growth and physiological parameters were measured at two sampling periods (35 DAT and 49 DAT) with six replicates per treatment and growth period. Plant height and leaf number were recorded. After harvest, upper fresh and dry biomass parts (leaves, petioles, leaf stem) and roots were measured. Different parts of the plants were separated to evaluate the uptake and translocation of Cu from the roots to upper plant parts and the relevant effects on nutrient accumulation. Individual samples were collected and put at 85 °C in a forced-air oven until constant weight was achieved to determine their dry weight. 

Leaf stomatal conductance was measured with a ΔT-Porometer AP4 (Delta-T Devices Cambridge, Burwell, Cambridge, UK) [66]. Leaf chlorophyll fluorescence (chlorophyll fluorometer, opti-sciences OS-30p, Hertfordshire, UK) was measured on two fully developed, light-exposed leaves per plant. Following leaf incubation in the dark for 20 min, the Fv/Fm ratio was measured [66]. Leaf chlorophyll was extracted with dimethyl sulfoxide (DMSO) and chlorophyll a (Chl a), chlorophyll b (Chl b) and total chlorophylls (total Chl) were assayed and expressed as μg g^−1^ FW [66].

### 4.3. Antioxidant Activity, Total Phenols and Total Flavonoids Content 

The antioxidant activity of the methanolic leaf plant extracts was determined with four replicates per treatment and sampling date by the assays of 2,2-diphenyl-1-picrylhydrazyl (DPPH) and ferric reducing antioxidant power (FRAP), as previously described by Chrysargyris et al. [67], as well as the 2,2′-azino-bis(3-ethylbenzothiazoline-6-sulphonic acid) (ABTS) assay according to the methodology described by Woidjylo et al. [68]. The Folin–Ciocalteu method was used for determining the total phenols content, as previously described [69] and results were expressed as gallic acid equivalents (mg GAE per g FW). The total flavonoid content was determined according to aluminum chloride colorimetric method [70] and results were expressed as rutin equivalents (mg rutin per g FW).

### 4.4. Plant Stress Indicators 

Cell damage index of lipid peroxidation in leaves was assessed in terms of malondialdehyde (MDA) content, which was determined by the thiobarbituric acid reaction [71]. Hydrogen peroxide (H_2_O_2_) content was measured according to the method of Loreto and Velikova [47]. The results were expressed as nmol MDA or μmol H_2_O_2_ per g FW. Four replicates were analyzed for each treatment and sampling date.

### 4.5. Nutrient Content

Dried tissue (0.5 g) from leaves, stems, leaf petioles and roots from each treatment (4 biological replications; each replication was a pool of 2 individual plants) at both sampling dates, was subjected to dry ashing at 450 °C and acid extraction (2N HCl). The extracts were used for the determination of sodium (Na) and potassium (K) by flame photometry (Lasany Model 1832, Lasany International, Panchkula, India), phosphorus (P) with the molybdate/vanadate method (yellow method) by spectrophotometry (Multiskan GO, Thermo Fischer Scientific, Waltham, MA, USA), zinc (Zn) and copper (Cu) by atomic absorption spectrometry (PG Instruments AA500FG, Leicestershire, UK). Nitrogen (N) was determined using the Kjeldahl method (BUCHI, Digest automat K-439 and Distillation Kjelflex K-360, Flawil, Switzerland) following Chrysargyris et al. [64]. In particular, the measured Cu content in this study refers to total dissolved Cu content, which was almost totally (≥98.21%) available as Cu^2+^ [49]. Plant nutrient content was expressed in g kg^−1^ and mg kg^−1^ DW, for macronutrients and micronutrients, respectively.

The Cu accumulation rate (AR), bioaccumulation coefficient (BAC), translocation factor (TF) and tolerance index (TI) of pelargonium were calculated by equations described by Benimeli et al. [72], Amin et al. [2] and Azooz et al. [73], as follows.

The accumulation rate (AR) was calculated as the sum up of Cu concentration in each plant tissue x plant DW divided by the number of days under Cu levels by the total plant DW [72].

(1) $$\begin{array}{c} \mathrm{Accumulation}\;\mathrm{rate}\;\mathrm{mg}\;\mathrm{per}\;(\mathrm{kg}\;\mathrm{DW}\;x\;\mathrm{day})=\\ \frac{(\left[\mathrm{Cu}\right]\;\mathrm{leave}\;x\;\mathrm{DW}\;\mathrm{leave}\;+\;(\left[\mathrm{Cu}\right]\;\mathrm{stem}\;x\;\mathrm{DW}\;\mathrm{stem}\;\;+\;(\left[\mathrm{Cu}\right]\;\mathrm{petiole}\;x\;\mathrm{DW}\;\mathrm{petiole}\;+\;\left(\left[\mathrm{Cu}\right]\;\mathrm{root}\;x\;\mathrm{DW}\;\mathrm{root}\right)}{\mathrm{Days}\;x\;\left(\mathrm{DW}\;\mathrm{leave}\;+\;\mathrm{DW}\;\mathrm{stem}\;+\;\mathrm{DW}\;\mathrm{petiole}\;+\;\mathrm{DW}\;\mathrm{root}\right)} \end{array}$$

The bioaccumulation coefficient (BAC) was calculated as the ratio of Cu concentration in plant tissue to that of Cu concentration in nutrient solution, according to Amin et al. [2]:

(2) $$\mathrm{Bioaccumulation}\;\mathrm{coefficient}\;=\frac{\mathrm{Cu}\;\mathrm{concentration}\;\mathrm{in}\;\mathrm{plant}\;\mathrm{tissue}\;\left(\mathrm{mg}\;\mathrm{kg}\;\mathrm{DW}\right)}{\mathrm{Cu}\;\mathrm{concentration}\;\mathrm{in}\;\mathrm{nutrient}\;\mathrm{solution}\;\left(\mathrm{mg}\;\mathrm{per}\;L\right)}$$

The translocation factor (TF) was calculated as the ratio of Cu concentration in plant tissue to that of Cu concentration in plant roots according to Amin et al. [2]:

(3) $$\mathrm{Translocation}\;\mathrm{factor}\;=\frac{\mathrm{Cu}\;\mathrm{concentration}\;\mathrm{in}\;\mathrm{plant}\;\mathrm{tissue}\;\left(\mathrm{mg}\;\mathrm{kg}\;\mathrm{DW}\right)}{\mathrm{Cu}\;\mathrm{concentration}\;\mathrm{in}\;\mathrm{plant}\;\mathrm{root}\;\left(\mathrm{mg}\;\mathrm{per}\;\mathrm{kg}\;\mathrm{DW}\right)}$$

Copper tolerance index (TI) was calculated as the quotient of the dry weight of plants grown under copper treated and control conditions according to the following the equations described by Benimeli et al. [72] and Azooz et al. [73], with the following modifications:

(4) $$\mathrm{Tolerance}\;\mathrm{index}\;(\%)\;=\frac{\mathrm{Dry}\;\mathrm{weight}\;\mathrm{of}\;\mathrm{Cu}-\mathrm{treated}\;\mathrm{plants}\;\times 100}{\mathrm{Dry}\;\mathrm{weight}\;\mathrm{of}\;\mathrm{Cu}-\mathrm{untreated}\;\mathrm{plants}\;\left(\mathrm{control}\right)}$$

### 4.6. Statistical Analysis

For plant growth and physiological measurements, six samples were used per treatment, whereas chemical composition/antioxidants were recorded from four samples per treatment. The analysis of the data was accomplished with the use of SPSS v. 22.0 program (IBM Corp., Armonk, NY, USA) and the one-way analysis of variance (ANOVA) was carried out for the Cu concentration for each sampling date, while means were compared with the Duncan multiple range test (DMRT) at *p* < 0.05, when significant differences were detected. Results were expressed as mean values and standard error (SE). The two-way ANOVA was also performed, with both Cu concentration and sampling date as the sources of variation. Finally, a regression analysis was applied to the content of Cu in plant tissues and the biochemical parameters associated with antioxidant response and oxidative stress.

## 5. Conclusions

Hydroponically grown *P. graveolens* resulted in a species tolerant toward high Cu concentrations in the root zone and the initial symptoms of Cu toxicity. Namely, declines of photosynthesis-related parameters and increases in leaf H_2_O_2_ along with considerable Cu accumulation in root tissues were evidenced only at the 100 μM Cu concentration in the nutrient solution. However, the extent of the toxicity symptoms did not have an impact on biomass production; in addition, high Cu levels stimulated plant secondary metabolism, enhancing the production of bioactive antioxidant molecules. Due to low Cu translocation to the aerial organs during the whole growing cycle, this microelement did not reach the leaf tissues, which resulted in suitable plant material for the safe extraction of bioactive compounds. These results show that plant stress from excess Cu does not necessarily preclude the use of MAPs for medicinal purposes, depending on the target organ where the metal accumulates. The outcome of this study showed that the leaves of *P. graveolens* plants exposed to excess Cu could be safely employed for their medicinal properties in herbal or pharmaceutical preparations.

## Figures and Tables

**Table 1:** Effect of increasing copper (Cu) concentration (4–25–50–100 μM Cu^+2^) in the nutrient solution and sampling date after transplanting (DAT, 35 days and 49 days) on plant height (cm), leaf number, total upper fresh biomass (g plant^−1^), and biomass dry matter content (%) in pelargonium plants grown hydroponically in perlite.

DAT	Cu^2+^ (μM)	Plant Height	Leaf Number	Total Upper Fresh Biomass	Total Upper Biomass Dry Matter Content
**35 days**	**4**	43.16 ± 2.19 ^Y^	32.50 ± 4.66 a	57.14 ± 3.78 ab	12.19 ± 0.48
	**25**	44.33 ± 1.72	24.66 ± 1.17 b	68.39 ± 2.75 a	12.19 ± 0.28
	**50**	42.33 ± 2.67	25.50 ± 3.52 b	70.02 ± 4.69 a	12.16 ± 0.91
	**100**	42.83 ± 2.15	22.33 ± 2.84 b	50.72 ± 6.10 b	13.16 ± 0.91
**49 days**	**4**	53.00 ± 4.57	58.00 ± 6.29	160.04 ± 0.80	12.89 ± 0.07 b
	**25**	50.00 ± 5.19	41.83 ± 6.87	152.92 ± 21.32	12.68 ± 0.15 b
	**50**	54.83 ± 9.22	42.33 ± 8.36	156.89 ± 10.77	13.77 ± 0.08 a
	**100**	56.40 ± 2.22	52.80 ± 4.66	133.55 ± 20.87	13.72 ± 0.19 a
***Significance***					
***Days (D)***		*ns*	*	***	*ns*
***Copper (Cu)***		*ns*	*ns*	*ns*	*ns*
***D x Cu***		*ns*	*ns*	*ns*	*ns*

^Y^ At each sampling date, values (*n* = 6) in columns followed by different letters are significantly different, *p* < 0.05, for each plant growth stage. *ns*, * and *** indicate non-significant or significant differences at *p* < 5%, and 0.1%, respectively, following two-way ANOVA.

**Table 2:** Effect of increasing copper (Cu) concentration (4–25–50–100 μM Cu^2+^) in the nutrient solution and sampling date after transplanting-DAT (35 days and 49 days) on leaf stomatal resistance (cm s^−1^), chlorophyll fluorescence (Fv/Fm), chlorophylls (Chl a, Chl b, Total Chl) content (μg g^−1^ fresh weight) in pelargonium plants grown hydroponically in perlite.

DAT	Cu^2+^ (μM)	Stomatal Resistance	Fv/Fm	Chl a	Chl b	Total Chl
**35 days**	**4**	0.90 ± 0.13 b ^Y^	0.83 ± 0.003 a	23.47 ± 0.35	36.32 ± 0.77	59.77 ± 1.04
	**25**	1.33 ± 0.08 ab	0.83 ± 0.001 a	23.80 ± 2.21	35.12 ± 2.57	58.90 ± 4.77
	**50**	1.34 ± 0.15 ab	0.83 ± 0.050 ab	23.97 ± 0.39	35.59 ± 0.71	59.54 ± 0.83
	**100**	1.61 ± 0.19 a	0.82 ± 0.003 b	23.49 ± 2.57	34.56 ± 2.15	58.03 ± 5.72
**49 days**	**4**	6.75 ± 0.50 b	0.80 ± 0.006 a	28.14 ± 1.10 a	39.76 ± 0.96 a	66.55 ± 1.39 a
	**25**	9.11 ± 1.20 ab	0.78 ± 0.005 ab	26.81 ± 0.43 ab	36.62 ± 0.14 ab	66.81 ± 2.60 a
	**50**	9.05 ± 0.78 ab	0.78 ± 0.007 ab	24.79 ± 0.85 b	38.68 ± 1.52 ab	66.81 ± 2.60 ab
	**100**	10.58 ± 1.38 a	0.75 ± 0.019 b	21.46 ± 0.91 c	35.22 ± 1.30 b	56.66 ± 2.17 b
***Significance***						
***Days (D)***		***	***	*ns*	*ns*	*ns*
***Copper (Cu)***		*	*ns*	*	*ns*	*ns*
***D x Cu***		*ns*	*ns*	*ns*	*ns*	*ns*

^Y^ At each sampling date, values (*n* = 6) in columns followed by different letters are significantly different, *p* < 0.05, for each plant growth stage. *ns*, * and *** indicate non-significant or significant differences at *p* < 5%, and 0.1%, respectively, following two-way ANOVA.

**Table 3:** Accumulation rate (AR, mg kg^−1^ DW day^−1^), bioaccumulation coefficient (BAC), and translocation factor (TF) for Cu after 35 and 49 DAT in pelargonium plants grown hydroponically in perlite.

DAT	Cu^2+^ (μM)	Accumulation Rate-AR (mg kg^−1^ DW day^−1^)	Bioaccumulation Coefficient (BAC)				Translocation Factor (TF)		
			Leaves	Stems	Petioles	Roots	Leaves	Stems	Petioles
**35 days**	**4**	94.94 ± 18.78 b ^Y^	140.66 ± 13.50 a	124.48 ± 4.71 a	104.66 ± 1.17 a	575.99 ± 90.69 a	0.25 ± 0.03 a	0.22 ± 0.03 a	0.19 ± 0.03 a
	**25**	145.97 ± 9.78 ab	24.03 ± 0.66 b	21.66 ± 0.64 b	19.34 ± 0.45 b	193.77 ± 15.76 b	0.12 ± 0.01 b	0.11 ± 0.01 b	0.10 ± 0.01 b
	**50**	165.14 ± 30.56 a	12.40 ± 0.14 b	11.49 ± 0.41 c	11.89 ± 0.24 c	84.51 ± 14.58 b	0.15 ± 0.03 b	0.14 ± 0.02 b	0.11 ± 0.03 b
	**100**	124.05 ± 12.80 ab	6.27 ± 0.14 b	5.89 ± 0.11 c	6.67 ± 0.13 d	73.67 ± 6.63 b	0.08 ± 0.01 b	0.08 ± 0.00 b	0.09 ± 0.01 b
**49 days**	**4**	549.13 ± 23.12	133.81 ± 6.98 a	104.82 ± 2.14 a	124.29 ± 6.70 a	212.23 ± 3.35 a	0.63 ± 0.02 a	0.49 ± 0.00 a	0.58 ± 0.03 a
	**25**	676.26 ± 170.78	23.60 ± 0.71 b	18.66 ± 2.62 b	19.89 ± 0.66 b	137.88 ± 4.70 b	0.17 ± 0.01 b	0.13 ± 0.02 b	0.14 ± 0.00 b
	**50**	965.19 ± 42.52	11.31 ± 0.72 c	9.96 ± 0.77 c	8.58 ± 0.62 c	124.42 ± 21.48 b	0.09 ± 0.02 c	0.08 ± 0.01 c	0.07 ± 0.01 c
	**100**	955.28 ± 260.27	6.01 ± 0.35 c	6.49 ± 0.45 c	4.37 ± 0.06 c	67.25 ± 0.92 c	0.09 ± 0.00 c	0.09 ± 0.01 c	0.06 ± 0.00 c
***Significance***									
***Days (D)***		***	*ns*	***	*	***	***	***	***
***Copper (Cu)***		*ns*	***	***	***	***	***	***	***
***D x Cu***		*ns*	*ns*	***	***	***	***	***	***

^Y^ At each sampling date, values (*n* = 6) in columns followed by different letters are significantly different, *p* < 0.05, for each plant growth stage. *ns*, *, and *** indicate non-significant or significant differences at *p* < 5%, and 0.1%, respectively, following two-way ANOVA.

**Table 4:** Tolerance indices (TI (%)) for Cu after 35 and 49 DAT in pelargonium plants grown hydroponically in perlite.

DAT	Cu^2+^ (μM)	Tolerance Indices-TI (%)										
		Total Biomass	Plant height	Leaf No	Leaf FW	Stem FW	Petiole FW	Root FW	Leaf DW	Stem DW	Petiole DW	Root DW
**35 days**	**4**	100.00 ± 0.00 ab ^Y^	100.00 ± 0.00	100.00 ± 0.00 a	100.00 ± 0.00 ab	100.00 ± 0.00 ab	100.00 ± 0.00 b	100.00 ± 0.00	100.00 ± 0.00 ab	100.00 ± 0.00	100.00 ± 0.00 b	100.00 ± 0.00
	**25**	111.30 ± 1.93 a	102.70 ± 3.98	75.89 ± 3.61 b	114.85 ± 0.12 a	117.05 ± 11.33 a	139.71 ± 4.48 a	113.49 ± 4.23	108.33 ± 1.82 a	110.93 ± 10.65	127.97 ± 1.03 a	98.97 ± 6.01
	**50**	113.84 ± 7.53 a	98.07 ± 6.20	78.46 ± 10.85 b	117.98 ± 8.13 a	120.51 ± 9.77 a	138.56 ± 6.94 a	93.54 ± 9.51	111.93 ± 8.57 a	109.79 ± 7.88	134.52 ± 2.06 a	117.89 ± 10.14
	**100**	88.31 ± 8.01 b	99.22 ± 4.98	68.71 ± 8.76 b	88.34 ± 10.83 b	81.90 ± 8.49 b	100.82 ± 23.47 b	91.53 ± 15.81	84.16 ± 10.72 b	87.92 ± 3.94	111.31 ± 7.21 b	93.33 ± 12.07
**49 days**	**4**	100.00 ± 0.00 b	100.00 ± 0.00	100.00 ± 0.00	100.00 ± 0.00	100.00 ± 0.00	100.00 ± 0.00	100.00 ± 0.00	100.00 ± 0.00	100.00 ± 0.00	100.00 ± 0.00	100.00 ± 0.00 b
	**25**	219.37 ± 77.36 a	94.33 ± 9.80	72.12 ± 11.84	92.36 ± 14.09	100.74 ± 12.76	91.83 ± 12.71	94.11 ± 3.02	89.62 ± 14.77	103.03 ± 13.16	91.55 ± 14.43	92.56 ± 1.19 b
	**50**	104.63 ± 6.51 b	103.45 ± 17.39	72.98 ± 14.42	96.47 ± 5.56	102.19 ± 8.24	92.66 ± 6.20	86.56 ± 10.47	98.95 ± 5.91	115.32 ± 7.77	101.20 ± 5.87	106.55 ± 13.72 ab
	**100**	89.36 ± 15.10 b	106.41 ± 4.20	91.03 ± 8.03	85.44 ± 14.69	83.03 ± 12.44	79.57 ± 10.38	98.23 ± 1.84	87.38 ± 15.64	93.41 ± 15.21	87.41 ± 12.84	124.10 ± 1.03 a
***Significance***												
***Days (D)***		*ns*	*ns*	*ns*	*ns*	*ns*	***	*ns*	*ns*	*ns*	***	*ns*
***Copper (Cu)***		*	*ns*	*ns*	*ns*	*	*	*ns*	*ns*	*ns*	*ns*	*ns*
***D x Cu***		*ns*	*ns*	*ns*	*ns*	*ns*	*	*ns*	*ns*	*ns*	*ns*	*ns*

^Y^ At each sampling date, values (*n* = 6) in columns followed by different letters are significantly different, *p* < 0.05, for each plant growth stage. *ns*, * and *** indicate non-significant or significant differences at *p* < 5%, and 0.1%, respectively, following two-way ANOVA.

**Table 5:** Pearson’s correlation table for leaf and root content of copper (Cu), leaf content of total phenols, flavonoids, H_2_O_2_ and malondialdehyde (MDA), and leaf antioxidant capacity determined using FRAP, DPPH or ABTS assays, in pelargonium plants grown hydroponically in perlite and exposed to four different Cu concentrations (4–25–50–100 μM Cu^2+^) in the nutrient solution, sampled at 35 and 49 days after transplanting (DAT).

	*Leaf Cu*	*Root Cu*	*Phenols*	*Flavonoids*	*FRAP*	*DPPH*	*ABTS*	*H*_2_ *O*_2_	*MDA*
**35 DAT**									
Leaf Cu	1								
Root Cu	0.8406	1							
Phenols	−0.3210	−0.0500	1						
Flavonoids	0.4770	−0.0714	−0.4142	1					
FRAP	0.4453	−0.0852	−0.2426	0.9835	1				
DPPH	0.5612	0.0339	−0.3562	0.9920	0.9866	1			
ABTS	0.5018	0.0037	−0.1406	0.9551	0.9901	0.9750	1		
H_2_O_2_	−0.3121	−0.0544	0.9996	−0.3893	−0.2161	−0.3310	−0.1139	1	
MDA	0.5334	0.1429	−0.9396	0.6778	0.5357	0.6432	0.4571	−0.9308	1
**49 DAT**									
Leaf Cu	1								
Root Cu	0.7078	1							
Phenols	0.8042	0.8743	1						
Flavonoids	0.7933	0.9364	0.9890	1					
FRAP	0.4427	0.9454	0.7185	0.8086	1				
DPPH	0.8973	0.3775	0.4626	0.4440	0.0855	1			
ABTS	0.7591	0.8380	0.9954	0.9751	0.6881	0.4034	1		
H_2_O_2_	0.4695	0.3948	0.7745	0.6815	0.2355	0.1705	0.8267	1	
MDA	−0.1303	−0.6349	−0.1807	−0.3241	−0.7818	0.0074	−0.1173	0.4215	1
